# The effect of sex and protein supplementation on bone metabolism during a 36-h military field exercise in energy deficit

**DOI:** 10.1152/japplphysiol.00106.2023

**Published:** 2023-05-04

**Authors:** Thomas J. O’Leary, Charlotte V. Coombs, Victoria C. Edwards, Sam D. Blacker, Rebecca L. Knight, Fiona N. Koivula, Jonathan C. Y. Tang, William D. Fraser, Sophie L. Wardle, Julie P. Greeves

**Affiliations:** ^1^Army Health and Performance Research, Army Headquarters, Andover, United Kingdom; ^2^Division of Surgery and Interventional Science, University College London, London, United Kingdom; ^3^Occupational Performance Research Group, University of Chichester, Chichester, United Kingdom; ^4^Norwich Medical School, University of East Anglia, Norwich, United Kingdom; ^5^Norfolk and Norwich University Hospital, Norwich, United Kingdom

**Keywords:** bone remodeling, energy availability, female athlete triad, stress fracture

## Abstract

This study investigated sex differences in, and the effect of protein supplementation on, bone metabolism during a 36-h military field exercise. Forty-four British Army Officer cadets (14 women) completed a 36-h field exercise. Participants consumed either their habitual diet [*n* = 14 women (Women) and *n* = 15 men (Men Controls)] or the habitual diet with an additional 46.6 g·day^−1^ of protein for men [*n* = 15 men (Men Protein)]. Women and Men Protein were compared with Men Controls to examine the effect of sex and protein supplementation. Circulating markers of bone metabolism were measured before, 24 h after (postexercise), and 96 h after (recovery) the field exercise. Beta C-telopeptide cross links of type 1 collagen and cortisol were not different between time points or Women and Men Controls (*P* ≥ 0.094). Procollagen type I N-terminal propeptide decreased from baseline to postexercise (*P* < 0.001) and recovery (*P* < 0.001) in Women and Men Controls. Parathyroid hormone (PTH) increased from baseline to post-exercise (*P* = 0.006) and decreased from postexercise to recovery (*P* = 0.047) in Women and Men Controls. Total 25(OH)D increased from baseline to postexercise (*P* = 0.038) and recovery (*P* < 0.001) in Women and Men Controls. Testosterone decreased from baseline to post-exercise (*P* < 0.001) and recovery (*P* = 0.007) in Men Controls, but did not change for Women (all *P* = 1.000). Protein supplementation in men had no effect on any marker. Men and women experience similar changes to bone metabolism—decreased bone formation and increased PTH—following a short-field exercise. Protein had no protective effect likely because of the energy deficit.

**NEW & NOTEWORTHY** Energy deficits are common in arduous military training and can cause disturbances to bone metabolism. This study provides first evidence that short periods of severe energy deficit and arduous exercise—in the form of a 36-h military field exercise—can suppress bone formation for at least 96 h, and the suppression in bone formation was not different between men and women. Protein feeding does not offset decreases in bone formation during severe energy deficits.

## Graphical abstract

**Figure:**
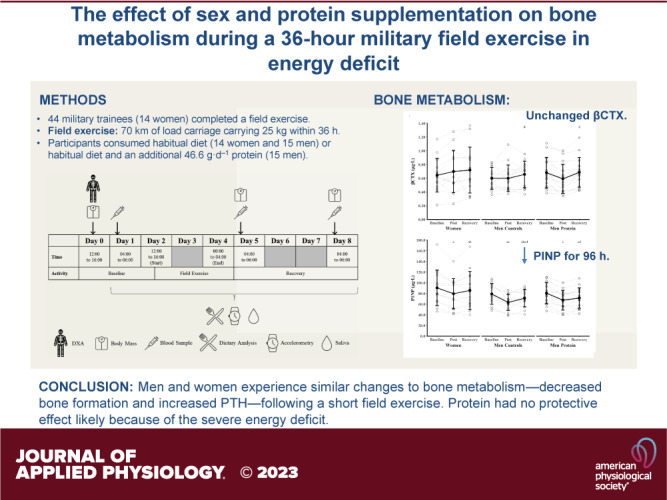

## INTRODUCTION

Military personnel are exposed to high exercise volumes and severe energy deficits (energy intake lower than total energy expenditure) during training courses and field exercises (1). Short periods of military training (from several days to 8 wk) in energy deficit result in endocrine changes in male soldiers—increased cortisol and decreased insulin-like growth factor-I (IGF-I), testosterone, estradiol, and thyroid hormones (2–9). There is some evidence that these endocrine disturbances lead to decreased markers of bone formation in men after 8 wk of training (8, 10), but evidence for the effects of acute periods (several days) of military field exercises on bone metabolism is limited, with even fewer data in women (1).

Acute periods (several days) of low energy availability (energy intake minus exercise energy expenditure) in women increase circulating markers of bone resorption and decrease circulating markers of bone formation (11, 12). Chronic low energy availability is associated with decreased areal bone mineral density and increased stress fracture risk, classically observed in female athletes (13). There is emerging evidence that male athletes experience similar endocrine and bone metabolic responses to low energy availability, although men may be more resistant to these metabolic effects than women (14); to our knowledge, only one study has compared the bone metabolic response to energy deficits in men and women (12). Women have recently been allowed to enter combat roles alongside men in the UK Armed Forces and other nations, but there is a lack of data on women examining the bone metabolic responses to the physiological stressors—high levels of physical activity, energy deficiency, and sleep deprivation—associated with combat training (1, 15). Alongside energy deficiency, sleep deprivation can also increase circulating markers of bone resorption and decrease circulating markers of bone formation (16), whereas exercise can increase markers of bone resorption and formation (17). The primary aim of this study was to investigate sex differences in markers of bone metabolism following a short arduous military field exercise. A better understanding of the effects of short periods of military field exercise, and subsequent recovery, on bone metabolism, will help develop strategies to protect skeletal health in operationally relevant settings and military training. We hypothesized that the field exercise would increase bone resorption and decrease bone formation—primarily due to the effects of energy deficiency—more in women than in men.

Evidence for the effect of additional dietary energy during military training in energy deficits on metabolic and endocrine markers is mixed (1). However, supplementary energy increased bone formation (8) and attenuated changes to the thyroid hormones (3) and IGF-I axis (8) but did not influence the changes in reproductive hormones (2, 3, 8). Although providing supplemental energy is one strategy to overcome energy deficits in military training, complete mitigation of energy deficits in this environment is difficult and impractical due to high total energy expenditures, limited time to eat or other logistical barriers, and suppressed appetite (1). Targeted specific macronutrient or micronutrient supplementation during energy deficits may help protect bone metabolism. Protein plays a structural role in the bone matrix, and protein feeding increases intestinal calcium absorption and may attenuate changes in concentrations of anabolic and metabolic hormones (18). Increasing protein intake during 8 to 10 days of military field exercise in energy deficit did not prevent changes in testosterone, thyroid hormones, or the IGF-I axis (19, 20), and a ∼40 g·day^−1^ protein supplement had no effect on markers of bone metabolism compared with a carbohydrate supplement during 9 wk of basic military training (21). There are limited data examining the effect of protein supplementation on bone metabolism in military training and no study has examined a short-term military field exercise in energy deficit. The secondary aim of this trial was to examine the effect of protein supplementation during a short and arduous field exercise on markers of bone metabolism in men. We hypothesized the field exercise would increase bone resorption and decrease bone formation, and supplementary protein would protect against these disturbances.

## METHODS

### Participants

Forty-five British Army Officer Cadets (15 women, 30 men) volunteered to take part in this mixed methods trial. All participants were recruited in July 2019 during *week 7* of their 44-wk British Army Officer Commissioning Course at the Royal Military Academy, Sandhurst, United Kingdom. The Officer Commissioning Course is a basic military training course comprising three 14-wk terms, each separated by 2 or 3 wk of leave, with 2 wk of adventure training following term two. The Officer Commissioning Course teaches soldiering skills and military leadership and is physically arduous. Officer Cadets complete aerobic and resistance training, military-specific fitness training, military drill, progressive loaded marching, learn basic military skills, and complete several arduous field exercises. The study was advertised to all women and men on the Officer Commissioning Course and the first 15 women and 30 men to volunteer were accepted into the study. The 15 women consumed the habitual diet (Women), whereas the 30 men were randomized (1:1) using block randomization to either the habitual diet (Men Controls) or the habitual diet with additional protein (Men Protein). The first part of this trial compared Women with Men Controls in an observational cohort study to examine sex differences in our outcomes. The second part of this trial was an unblinded randomized controlled trial with a parallel group design, whereby Men Controls with Men Protein were compared to examine the effect of protein supplementation on our outcomes. The low numbers of women in British Army Officer training (∼25 women and ∼200 men in each course) meant it was not possible to randomize a group of women to be supplemented with protein. All participants passed an initial military medical assessment and were confirmed injury free and medically fit before starting training. Exclusion criteria for entry to the military were pregnancy; adrenal, ovarian, or gonadotropin-releasing hormone insufficiency; pituitary disease; thyroid disease in the past year; diabetes; hyperparathyroidism; osteopenia; glucocorticoid use; or musculoskeletal injury. Each participant had the study procedures and risks fully explained verbally and in writing before providing written informed consent. This study was approved by the Ministry of Defense Research Ethics Committee (Ref: 931/MoDREC/18).

### Experimental Design

**Figure 1.**
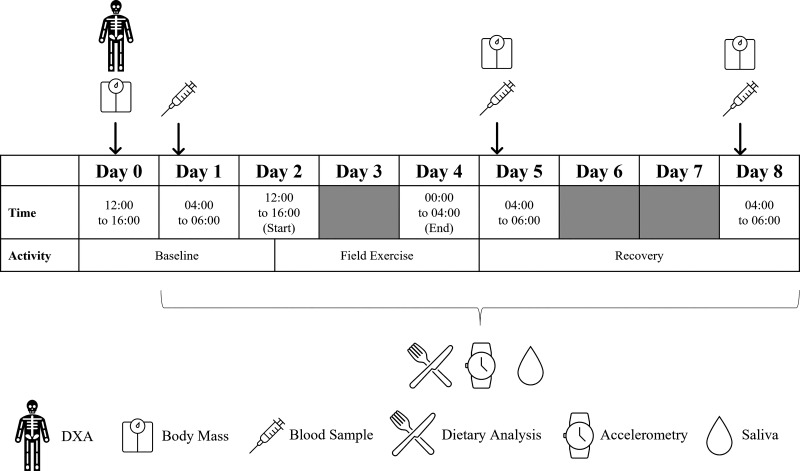
Overview of study design. DXA, dual-energy X-ray absorptiometry.

All participants completed a 36-h field exercise in the Brecon Beacons, Wales, UK, during *week 8* of their training course. The first 7 wk of military training involves a progressive increase in physical training intensity volume and intensity in the camp where sleep and food intake are protected. The field exercise consisted of completing ∼70 km of load carriage carrying 25 kg in a rucksack, helmet, and rifle across undulating and hilly terrain in teams of six. The 70-km course required each team of six to pass through 12 checkpoints within 36 h with ≤ 4-h sleep. Each team had a staggered start and finish to the field exercise resulting in all participants completing the field exercise over a ∼40-h period. Each team could pass the checkpoints in any order. Participants were enforced to take a 4-h break where they had the opportunity to sleep after 24 h. Each checkpoint required a team of six to complete a leadership or problem-solving task, and the checkpoints could be completed in any order as decided by each team. Total distance and elevation were recorded by GPS worn by one member of each team of six. One woman, one man in the control group, and one man supplemented with protein were part of each group of six to control for differences in the self-selected route. Following the field exercise, participants returned to normal training in camp where they were permitted to sleep between 2200 and 0600 h. Venous blood samples were drawn ∼18 h before (baseline), ∼24 h (postexercise), and 96 h (recovery) after the field exercise and analyzed for biochemical markers of bone formation, bone resorption, calcium metabolism, and reproductive and adrenal hormones (Fig. 1). A follow-up time of 96 h of recovery was chosen because participants had a break from military training following the end of the field exercise with a resumption of training after 96 h. Body mass was measured by calibrated scales at all time points. Whole body lean and fat mass were measured by dual-energy X-ray absorptiometry (DXA) at baseline. Energy expenditure was measured by accelerometry and using the doubly labeled water method. Energy intake was measured from food diaries when eating in camp and food wrappers and discards from the ration pack when on field exercise.

### Dietary Intervention and Dietary Assessment

Participants ate ad libitum from the military canteen when not on field exercise and ate from an operational ration pack during the field exercise. Participants could also supplement their diet with their own food at any time. The operational ration pack provides 4,000 kcal·day^−1^ in the form of ready-to-eat meals and snacks. The men supplemented with protein were provided an additional two protein-rich bars (217 kcal, 23.3 g protein, 13.6 g carbohydrate, and 8.2 g fat per bar) to consume per day throughout the trial. Dietary intake was measured by food diaries and the collection of all wrappers (including the protein-rich bars) for the 7 days of the trial. During the field exercise, participants carried the food diaries as part of their kit and recorded consumed items whenever they stopped to eat. Investigators were placed at 4 of the 12 checkpoints to assist with the collection of discards from the ration packs and any food wrappers. Nutritional intake was calculated for the 24 h before the field exercise (baseline), for the 48 h that included the field exercise (field exercise), and for the 96 h after the field exercise (recovery). Absolute energy, carbohydrate, protein, and fat intake were determined using Nutritics software (Nutritics, Ireland) and calculated as the mean per day for each of the three monitoring periods. Relative values were also calculated by dividing the absolute values by the body weight measured at that time point.

### Energy Expenditure

Total energy expenditure was estimated using a wrist-worn triaxial accelerometer (GENEActiv, Activinsights, UK). Participants were instructed to wear the accelerometers at all times. The accelerometers were set at a sampling frequency of 50 Hz and calibrated to each participant’s sex, age, height, and body mass. Raw acceleration data were analyzed to estimate metabolic equivalents (METs) using proprietary software (Activinsights, UK) and summed to calculate MET minutes (MET·min^−1^). Minutes with a zero value were replaced with 0.9 METs to reflect resting metabolism. Daily data were excluded if the device was worn <65% of the day. Total daily energy expenditure was calculated as MET.min × 3.5 × body mass (kg)/200 with an adjustment applied using a previously developed equation validated against doubly labeled water in a military training population: 563.116 + (0.886 × total daily energy expenditure) (22). Total energy expenditure was calculated for the 24 h before the field exercise (baseline), for the 48 h that included the field exercise (field exercise), and for the 96 h after the field exercise (recovery).

Total energy expenditure was measured using the doubly labeled water method (23). Following a baseline saliva sample, participants consumed a single-weighed oral dose of deuterium (^2^H) and oxygen-18 (^18^O) before a 7-day measurement period. Daily saliva samples were then collected at ∼0700 h for the following 7 days and stored at 4°C until analysis. Saliva samples were analyzed by isotope ratio mass spectrometry for the determination of rCO_2_. A food quotient was calculated for each participant from the dietary assessment data and used to estimate energy expenditure from rCO_2_ (23). Total energy expenditure was calculated for the total 7-day period. Absolute total energy expenditure values—measured from both accelerometry and doubly labeled water—were also converted to relative values by dividing by the body weight measured at the same time-point.

### Biochemical Markers of Bone Formation, Bone Resorption, and Calcium Metabolism

Venous blood was drawn from a vein in the antecubital fossa between 0400 and 0600 after an overnight fast from 2200 h. Serum separator vacutainers and EDTA vacutainers were stood at room temperature for 30 min before being centrifuged (Becton Dickinson) at 2,000 *g* at 4°C for 10 min. Serum and plasma were fractioned and stored at −80°C until analysis. Plasma samples were analyzed for procollagen type I N-terminal propeptide (PINP), c-terminal cross links telopeptide of type 1 collagen (βCTX), and intact parathyroid hormone (PTH) by electrochemiluminescence immunoassay on Cobas e601 platform (Roche Diagnostics, Germany) with interassay CVs of < 5.0% across their respective analytical ranges. Plasma testosterone and cortisol were analyzed by liquid chromatography-tandem mass spectrometry (LC-MS/MS) calibrated using commercial standards (Chromsystems, München, Germany) traceable to standard reference material SRM971 from the National Institute of Science and Technology (NIST). Plasma testosterone and cortisol had an interassay CV < 6.0% across the working range of 0.1– 39.9 nmol·L^−1^ and 0.1–806.0 nmol·L^−1^, respectively. Serum samples were analyzed for 25-hydroxyvitamin D (25(OH)D3 and 25(OH)D2) and 24,25-dihydroxyvitamin D (24,25(OH)_2_D3 and 24,25(OH)_2_D2) by LC-MS/MS and calibrated using standard reference material SRM972a from NIST. Total 25(OH)D and total 24,25(OH)_2_D were calculated from the sum of the measurements of D3 and D2 forms with an interassay CV < 10.0% across the working range of 0.1–200.0 nmol·L^−1^ and 0.1–30.0 nmol·L^−1^, respectively. Total 1,25-dihydroxyvitamin D (1,25(OH)_2_D) was analyzed by the DiaSorin LIAISON XL 1,25(OH)_2_D chemiluminescent immunoassay (Stillwater, MN) with an interassay CV ≤ 9.2% across the working range of 12–480 pmol·L^−1^. Serum total calcium, albumin, and phosphate were measured by spectrophotometric methods on the Cobas c501 platform (Roche Diagnostics, Germany) according to the manufacturer’s instructions with interassay CVs ≤ 2.1% across the working ranges of 0.20–5.00 mmol·L^−1^, 2–60 g·L^−1^, and 0.81–1.45 mmol·L^−1^, respectively. Albumin-adjusted calcium was calculated as = −0.8 × [albumin] − 4) + [total calcium]. All biochemical analysis was undertaken by the Good Clinical Laboratory Practice-certified Bioanalytical Facility at the University of East Anglia. All analytical processes meet the requirements specified by external national quality assurance schemes.

### Body Composition

Whole body lean mass and fat mass were assessed by DXA (Lunar iDXA, GE Healthcare, UK) at baseline (2 days before the field exercise) with participants wearing shorts and a T-shirt. Body mass was measured with calibrated scales (SECA, UK).

### Statistical Analyses

All data were analyzed using the R programming language (v.4.2.0). A minimum of 13 women and 13 men were necessary to detect a sex × time interaction for βCTX (η_p_^2^ = 0.04) (24) with an ɑ of 0.05, 1 - β of 0.80, and correlation among repeated measures of 0.7 (G*Power, v.3.1.9.2). Distribution of the data was checked using Shapiro–Wilk tests and frequency distribution histograms. Participant demographics were compared between Women and Men Controls with independent samples *t* tests or a Welch’s *t* test for groups with unequal variances; Men Controls and Men Protein were randomized to group and so were not compared. Field trial characteristics, total energy expenditure (doubly labeled water), and energy balance (doubly labeled water) were compared between Women and Men Controls and Men Controls and Men Protein with independent samples *t* tests or a Welch’s *t* test for groups with unequal variances. Linear mixed-effect models with restricted maximum likelihood estimation were used to examine changes in energy intake, carbohydrate intake, fat intake, protein intake, energy expenditure (accelerometry), energy balance (accelerometry), βCTX, PINP, PTH, albumin-adjusted calcium, phosphate, total 25(OH)D, total 1,25(OH)D_2_ total 24,24(OH)D_2_, cortisol, and testosterone (*lme4 package* v1.1.29). Separate linear mixed-effects models were run to examine the effect of sex and the effect of protein supplementation. Sex (Women vs. Men Controls), time (baseline vs. postexercise vs. recovery), and their interaction were included as fixed effects to examine sex differences. Group (Men Controls vs. Men Protein), time (baseline vs. postexercise vs. recovery), and their interaction were included as fixed effects to examine the effect of protein supplementation. The comparison of Men Protein with Men Controls was made with an intention-to-treat analysis. Random intercepts were assigned to each participant to account for within-participant correlation for repeated measures. Significance of the fixed effects from each model was determined with Satterthwaite degrees of freedom (*lmerTest package* v.3.1.3). Normality of the residuals for each model was checked visually by plotting the residuals against the fitted values and from Q–Q plots. In the event of a significant main effect of time or significant interaction, pairwise comparisons with Bonferroni corrections and Kerward-Roger degrees of freedom were used on the linear mixed-effects model to identify differences between time points or group (*emmeans package* v.1.7.3). Pooled data were used for main effects when there was no significant interaction, and each group was analyzed independently when there was a significant interaction. Effect sizes are presented as partial eta-squared (η_p_^2^) for main and interaction effects, Hedges’ *g* for between-group comparisons, and paired Hedges’ *g* for within-group paired comparisons (*effectsize package* v.0.6.0.1). Figures were drawn in the *ggplot2 package* (v.3.3.5). Significance was accepted as *P* ≤ 0.05.

## RESULTS

### Participants

**Figure 2.**
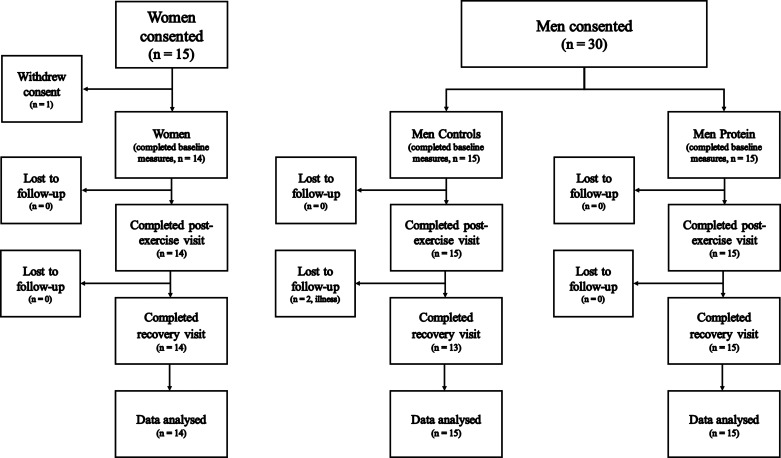
Participant flow through the study. Women were compared with Men Controls to examine sex differences. Men Controls were compared with men supplemented with protein (Men Protein) to examine the effects of protein supplementation.

Participant flow through the study is shown in Fig. 2. One woman withdrew consent before baseline measures, and two men from Men Controls were unavailable for blood samples at the recovery time point due to illness. Nutritional intake data were missing for five observations across four participants due to incomplete food diaries. Energy expenditure data estimated from accelerometers were missing for 20 observations across seven participants due to insufficient wear time. Total energy expenditure data measured by doubly labeled water data were missing for four Women, five Men, and three Men Protein due to missing saliva samples. There were no differences between Women and Men Controls for age (*P* = 0.670, *g* = 0.16), total 25(OH)D (*P* = 0.691, *g* = 0.14), or fat mass (*P* = 0.711, *g* = 0.14) but Women were shorter, lighter, and had less lean mass than Men Controls (all *P* < 0.001, *g* ≥ 2.15) (Table 1). There was no difference between Women and Men Controls (*P* ≥ 0.878, *g* ≤ 0.06) or Men Protein and Men Controls (*P* ≥ 0.645, *g* ≤ 0.17) for distance covered, elevation gain, or completion time during the field exercise (Table 1).

**Table 1.** Participant demographics and field exercise characteristics

*n*	Women 14	Men Controls 15	Men Protein 15
Age, yr	23 ± 1	23 ± 2	25 ± 3
Height, m	1.66 ± 0.07^a^	1.81 ± 0.07	1.84 ± 0.08
Body mass, kg	61.6 ± 6.6^a^	81.4 ± 7.9	84.4 ± 12.5
Lean mass, kg	45.3 ± 5.4^a^	63.5 ± 5.8	66.8 ± 8.5
Fat mass, kg	14.2 ± 2.4	14.6 ± 3.3	14.0 ± 4.8
Total 25(OH)D, nmol·L^−1^	73.2 ± 8.1	71.1 ± 17.5	80.5 ± 11.6
Distance, km	67.5 ± 12.4	66.8 ± 12.2	67.2 ± 11.9
Elevation gain, m	4,486 ± 1,158	4,424 ± 1,141	4,350 ± 1,100
Completion time, h:min	33:53 ± 3:00	33:52 ± 2:53	34:20 ± 2:39

Data are means (SD). ^a^*P* < 0.05 vs. men controls.

### Sex Differences in Nutritional Intake

Nutritional intake for Women and Men Controls is displayed in Table 2. Absolute and relative energy intake, absolute protein intake, and relative fat intake were not different between time points (main effects of time, *P* ≥ 0.173, η_p_^2^ ≤ 0.07) or Women and Men Controls (main effects of sex, *P* ≥ 0.093, η_p_^2^ ≤ 0.10; sex × time interaction, *P* ≥ 0.105, η_p_^2^ ≤ 0.09). There was a main effect of time for absolute carbohydrate intake (*P* = 0.004, η_p_^2^ = 0.19), but no difference between Women and Men Controls (main effect of sex, *P* = 0.314, η_p_^2^ = 0.04; sex × time interaction, *P* = 0.455, η_p_^2^ = 0.03). Absolute carbohydrate intake was lower in recovery than in baseline (*P* = 0.005, *g* = 1.12) and field exercise (*P* = 0.043, *g* = 0.39), with no difference between baseline and field exercise (*P* = 1.000, *g* = 0.24). There was a main effect of time for relative carbohydrate intake (*P* = 0.016, η_p_^2^ = 0.15), but Women and Men Controls changed similarly (sex × time interaction, *P* = 0.795, η_p_^2^ < 0.01). Relative carbohydrate intake was lower in recovery than in baseline (*P* = 0.021, *g* = 0.63), with no difference between baseline (*P* = 1.000, *g* = 0.12) or recovery (*P* = 0.071, *g* = 0.36) with field exercise. Relative carbohydrate intake was higher in Women than in Men Controls (main effect of group, *P* = 0.047, η_p_^2^ = 0.14). Absolute fat intake was not different between time points (main effect of time, *P* = 0.193, η_p_^2^ = 0.06; sex × time interaction, *P* = 0.658, η_p_^2^ = 0.02), but was lower in Women than in Men Controls (main effect of sex, *P* = 0.038, η_p_^2^ = 0.15). Relative protein intake was not different between time points (main effect of time, *P* = 0.759, η_p_^2^ < 0.01; sex × time interaction, *P* = 0.062, η_p_^2^ = 0.07), but was higher in Women than in Men Controls (main effect of sex, *P* = 0.033, η_p_^2^ = 0.06).

**Table 2.** Body mass, energy balance, and macronutrient intake

	Women (*n* = 14)				Men Controls (*n* = 15)				**Men Protein** (*n* = 15)			
	Baseline	Exercise*	Recovery	Total	Baseline	Exercise*	Recovery	Total	Baseline	Exercise*	Recovery	Total
Body mass, kg	61.6 ± 6.6^g^	60.8 ± 7.2^g^	61.5 ± 7.2^g^		81.4 ± 7.9	79.8 ± 8.3	81.4 ± 6.5		84.4 ± 12.5	84.2 ± 12.4	83.0 ± 14.6	
Energy intake												
Absolute, kcal·day^−1^	3,202 ± 1,013	3,007 ± 1,040	2,891 ± 725	2,924 ± 649	3,737 ± 770	3,612 ± 1,543	3,145 ± 804	3,296 ± 714	4,363 ± 866	5,006 ± 2,153	3,916 ± 1,219	4,189 ± 848^g^
Relative, kcal·kg·day^−1^	52 ± 15	49 ± 13	48 ± 11	48 ± 9	46 ± 10	45 ± 20	41 ± 9	42 ± 10	52 ± 9	61 ± 27	48 ± 13	50 ± 9^g^
Carbohydrate intake												
Absolute, g·day^−1^	376 ± 133	374 ± 130	321 ± 103^a,b^	335 ± 89	439 ± 96	415 ± 148	319 ± 80^a,b,c,d^	357± 77	493 ± 138	546 ± 235	382 ± 163^c,d^	431 ± 115^g^
Relative, g·kg·day^−1^	6.1 ± 2.1	6.2 ± 1.8	5.3 ± 1.6^a^	5.5 ± 1.3^g^	5.5 ± 1.4	5.2 ± 2.0	4.1 ± 1.0^a,c,d^	4.5 ± 1.1	5.9 ± 1.4	6.6 ± 2.9	4.6 ± 1.7^c,d^	5.2 ± 1.2
Fat intake												
Absolute, g·day^−1^	125 ± 51	115 ± 49	111 ± 38	112 ± 34^g^	156 ± 30	152 ± 84	126 ± 36^d^	135 ± 31	176 ± 47	210 ± 98	152 ± 61^d^	167 ± 39^g^
Relative, g·kg·day^−1^	2.0 ± 0.8	1.9 ± 0.6	1.9 ± 0.5	1.9 ± 0.4	1.9 ± 0.4	1.9 ± 1.1	1.7 ± 0.4	1.8 ± 0.4	2.1 ± 0.5	2.6 ± 1.3	1.8 ± 0.7	2.1 ± 0.4
Protein intake												
Absolute, g·day^−1^	129 ± 34	109 ± 40	98 ± 29	103 ± 26	112 ± 41	132 ± 73	126 ± 44	122 ± 35	172 ± 48	211 ± 91	160 ± 35	172 ± 21^g^
Relative, g·kg·day^−1^	2.1 ± 0.5	1.8 ± 0.6	1.6 ± 0.4	1.7 ± 0.3^g^	1.4 ± 0.5	1.7 ± 0.9	1.6 ± 0.6	1.6 ± 0.5	2.1 ± 0.6	2.6 ± 1.2	2.0 ± 0.5	2.1 ± 0.3^g^
Accelerometry												
Absolute EE, kcal·day^−1^	2,473 ± 722^h^	5,087 ± 915^e,h^	2,496 ± 692^f,h^	3,244 ± 729	3,460 ± 265	6,697 ± 542^c,e^	3,514 ± 296^d,f^	4,373 ± 459	3,818 ± 596	7,193 ± 951^c^	3,933 ± 574^d^	4,895 ± 677
Relative EE, kcal·kg·day^−1^	40 ± 11	82 ± 11^a^	41 ± 10^b^	53 ± 10	44 ± 4	87 ± 7a^,c^	44 ± 4^b,d^	55 ± 7	45 ± 4	86 ± 4^c^	48 ± 5^d^	59 ± 4
Absolute EB, kcal·day^−1^	764 ± 1,261	−1,998 ± 1,359^a^	439 ± 949^b^	−281 ± 952^g^	220 ± 587	−2,870 ± 1,699^a,c^	−433 ± 713^b,d^	−1,121 ± 562	545 ± 894	−2,187 ± 2,508^c^	−17 ± 989^d^	−706 ± 741
Relative EB, kcal·kg·day^−1^	12 ± 20	−33 ± 22^a^	7 ± 16^b^	−5 ± 15	3 ± 7	−36 ± 21^a,c^	−4 ± 7^b^	−13 ± 7	7 ± 11	−25 ± 28^c^	0 ± 12^d^	−8 ± 9
Doubly labeled water												
Absolute EE, kcal·day^−1^				3,557 ± 1,299				3,998 ± 1,242				5,159 ± 1,395
Relative EE, kcal·kg·day^−1^				60 ± 25				50 ± 15				64 ± 20
Absolute EB, kcal·day^−1^				−762 ± 1,304				−415 ± 1,068				−1,033 ± 1,407
Relative EB, kcal·kg·day^−1^				−13 ± 23				−5 ± 13				−13 ± 19

Data are means (SD). ^a^*P* < 0.05 vs. baseline (main effects, women and men controls pooled); ^b^*P* < 0.05 vs. exercise (main effects, women and men controls pooled); ^c^*P* < 0.05 vs. baseline (main effects, men controls and men protein pooled); ^d^*P* < 0.05 vs. exercise (main effects, men controls and men protein pooled); ^e^*P* < 0.05 vs. baseline (within group); ^f^*P* < 0.05 vs. exercise (within group); ^g^*P* < 0.05 vs. men controls (main effect of group); ^h^*P* < 0.05 vs. men controls (post hoc).

*Post exercise for body mass only.

EB, energy balance; EE, energy expenditure; total, the average of the total 7-day period.

### Sex Differences in Energy Balance

Energy expenditure and energy balance data for Women and Men Controls are displayed in Table 2. Body mass was not different between time points (main effect of time, *P* = 0.106, η_p_^2^ = 0.08; sex × time interaction, *P* = 0.623, η_p_^2^ = 0.02), but was higher in Men than in Women (main effect of sex, *P* < 0.001, η_p_^2^ = 0.67). There was a sex × time interaction for absolute accelerometry-estimated energy expenditure (*P* < 0.001, η_p_^2^ = 0.27). Absolute accelerometry-estimated energy expenditure increased from baseline to field exercise (*P* < 0.001, *g* ≥ 4.48) and decreased from field exercise to recovery (*P* < 0.001, *g* ≥ 5.55), with baseline and recovery not different (*P* = 1.000, *g* ≤ 0.13) in Women and Men Controls; the increase from baseline to field exercise was lower in Women than in Men Controls. Absolute accelerometry-estimated energy expenditure was lower in Women than in Men Controls at all time points (*P* < 0.001, *g* ≥ 1.75). There was a main effect of time for relative accelerometry-estimated energy expenditure and relative accelerometry-estimated energy balance (*P* < 0.001, η_p_^2^ ≥ 0.98), but no difference between Women and Men Controls (main effect of sex, *P* ≥ 0.134, η_p_^2^ ≤ 0.09; sex × time interaction, *P* ≥ 0.583, η_p_^2^ ≤ 0.03). Relative accelerometry-estimated energy expenditure increased from baseline to field exercise (*P* < 0.001, *g* = 6.41) and decreased from field exercise to recovery (*P* < 0.001, *g* = 7.75), with baseline and recovery not different (*P* = 1.000, *g* < 0.01). Relative accelerometry-estimated energy balance decreased from baseline to field exercise (*P* < 0.001, *g* = 1.89) and increased from field exercise to recovery (*P* < 0.001, *g* = 1.82), with baseline and recovery not different (*P* = 0.670, *g* = 0.36). There was a main effect of time for absolute accelerometry-estimated energy balance (*P* < 0.001, η_p_^2^ = 0.75), but Women and Men Controls changed similarly (sex × time interaction, *P* = 0.890, η_p_^2^ = 0.01). Absolute accelerometry-estimated energy balance decreased from baseline to field exercise (*P* < 0.001, *g* = 1.79) and increased from field exercise to recovery (*P* < 0.001, *g* = 1.71), with baseline and recovery not different (*P* = 0.398, *g* = 0.64). Absolute accelerometry-estimated energy balance was higher in Women than in Men Controls (main effect of sex, *P* = 0.038, η_p_^2^ = 0.17). Absolute and relative total energy expenditure and energy balance measured by doubly labeled water were not different between Women and Men Controls (*P* ≥ 0.296, *g* ≤ 0.49).

### The Effect of Protein Supplementation on Nutritional Intake

Nutritional intake for Men Controls and Men Protein can be seen in Table 2. Absolute and relative energy intake and protein intake were not different between time points (main effect of time, *P* ≥ 0.076, η_p_^2^ ≤ 0.09; group × time interaction, *P* ≥ 0.352, η_p_^2^ ≤ 0.04), but were higher in Men Protein than in Men Controls (main effect of group, *P* ≤ 0.018, η_p_^2^ ≥ 0.18). There was a main effect of time (*P* ≤ 0.037, η_p_^2^ ≥ 0.20) and group (*P* ≤ 0.025, η_p_^2^ ≥ 0.16) for absolute carbohydrate and absolute fat intake, but no group × time interactions (*P* ≥ 0.449, η_p_^2^ ≤ 0.03). Absolute carbohydrate intake was lower in recovery than in baseline (*P* = 0.011, *g* = 0.80) and field exercise (*P* = 0.006, *g* = 0.52), with no difference between baseline and field exercise (*P* = 1.000, *g* = 0.03). Absolute fat intake decreased from field exercise to recovery (*P* = 0.037, *g* = 0.39), but baseline and field exercise (*P* = 1.000, *g* = 0.18) and baseline and recovery (*P* = 0.287, *g* = 0.40) were not different. Absolute carbohydrate and absolute fat intake were higher in Men Protein than in Men Controls. There was a main effect of time for relative carbohydrate intake (*P* = 0.003, η_p_^2^ = 0.11), but no difference between Men Protein and Men Controls (main effect of group, *P* = 0.077, η_p_^2^ = 0.11; group × time interaction, *P* = 0.513, η_p_^2^ = 0.02). Relative carbohydrate intake was lower in recovery than in baseline (*P* = 0.018, *g* = 0.77) and field exercise (*P* = 0.006, *g* = 0.53), but baseline and field exercise were not different (*P* = 1.000, *g* = 0.05). Relative fat intake was not different between time points (main effect of time, *P* = 0.064, η_p_^2^ = 0.10) or Men Controls and Men Protein (main effect of group, *P* = 0.075, η_p_^2^ = 0.11; group × time interaction, *P* = 0.406, η_p_^2^ = 0.03).

### The Effect of Protein Supplementation on Energy Balance

Energy expenditure and energy balance data for Men Controls and Men Protein are displayed in Table 2. Body mass was not different between time points (main effect of time, *P* = 0.393, η_p_^2^ = 0.03) or Men Controls and Men Protein (main effect of group, *P* = 0.438, η_p_^2^ = 0.02; group × time interaction, *P* = 0.175, η_p_^2^ = 0.06). There was a main effect of time for absolute and relative accelerometry-estimated energy expenditure and energy balance (*P* < 0.001, η_p_^2^ ≥ 0.43), but no difference between Men Protein and Men Controls (main effect of group, *P* ≥ 0.066, η_p_^2^ ≤ 0.13; group × time interaction, *P* ≥ 0.058, η_p_^2^ ≤ 0.12). Absolute and relative accelerometry-estimated energy expenditure increased from baseline to field exercise (*P* < 0.001, *g* ≥ 6.01) and decreased from field exercise to recovery (*P* < 0.001, *g* ≥ 5.76), but baseline and recovery were not different (*P* = 1.000, *g* ≤ 0.30). Absolute and relative accelerometry-estimated energy balance decreased from baseline to field exercise (*P* < 0.001, *g* ≥ 1.12) and increased from field exercise to recovery (*P* < 0.001, *g* ≥ 0.91), but baseline and recovery were not different (*P* ≥ 0.449, *g* ≤ 0.43). Absolute and relative total energy expenditure and energy balance measured by doubly labeled water were not different between Men Protein and Men Controls (*P* ≥ 0.052, *g* ≤ 0.84).

### Sex Differences in Biochemical Markers of Bone Resorption, Bone Formation, and Calcium Metabolism

**Figure 3.**
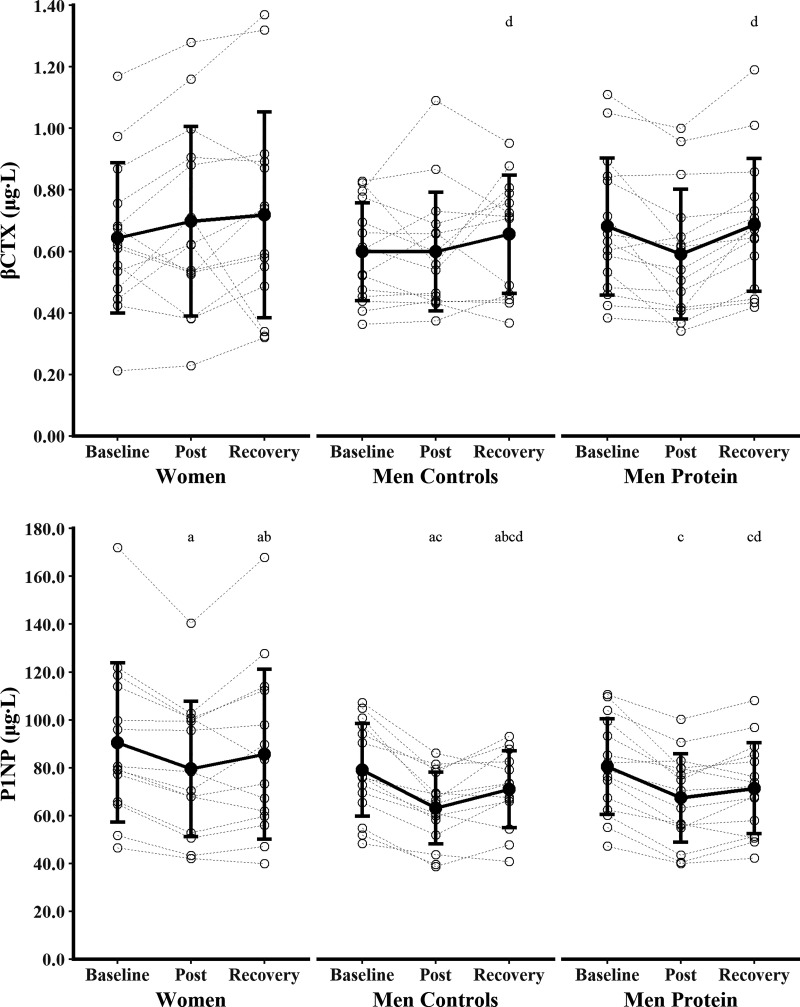
Biochemical markers of bone resorption (*top*) and bone formation (*bottom*) before (baseline), 24 h after (post), and 96 h after (recovery) the field exercise. Women (*n* = 14) and men supplemented with protein (Men Protein, *n* = 15) were independently compared with non-supplemented men (Men Controls, *n* = 15) to examine the effect of sex and protein supplementation. Data were analyzed with linear mixed-effects models. ^a^*P* < 0.05 vs. baseline (main effects, Women and Men Controls pooled); ^b^*P* < 0.05 vs. post (main effects, Women and Men Controls pooled); ^c^*P* < 0.05 vs. baseline (main effects, Men Controls and Men Protein pooled); ^d^*P* < 0.05 vs. post (main effects, Men Controls and Men Protein pooled). βCTX, beta C-telopeptide cross-links of type 1 collagen; PINP, procollagen I N-terminal propeptide.

**Figure 4.**
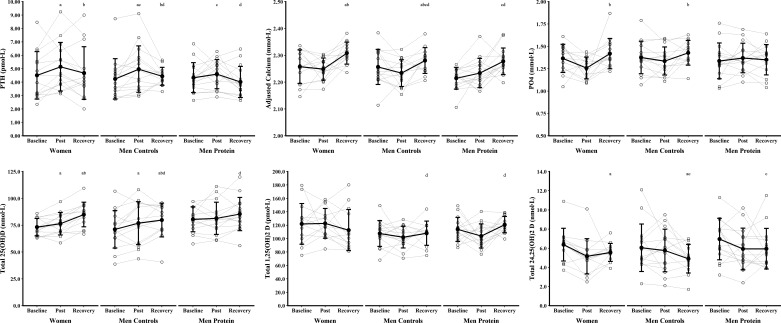
Biochemical markers of calcium metabolism before (baseline), 24 h after (post), and 96 h after (recovery) the field exercise. Women (*n* = 14) and men supplemented with protein (Men Protein, *n* = 15) were independently compared with nonsupplemented men (Men Controls, *n* = 15) to examine the effect of sex and protein supplementation. Data were analyzed with linear mixed-effects models. ^a^*P* < 0.05 vs. baseline (main effects, Women and Men Controls pooled); ^b^*P* < 0.05 vs. post (main effects, Women and Men Controls pooled); ^c^*P* < 0.05 vs. baseline (main effects, Men Controls and Men Protein pooled); ^d^*P* < 0.05 vs. post (main effects, Men Controls and Men Protein pooled). PO_4_, phosphate; PTH, parathyroid hormone; total 25(OH)D, total 25-hydroxyvitamin D; total 24,25(OH)_2_D, total 24,25-dihydroxyvitamin D; total 1,25(OH)_2_D, total 1,25-dihydroxyvitamin D.

**Figure 5.**
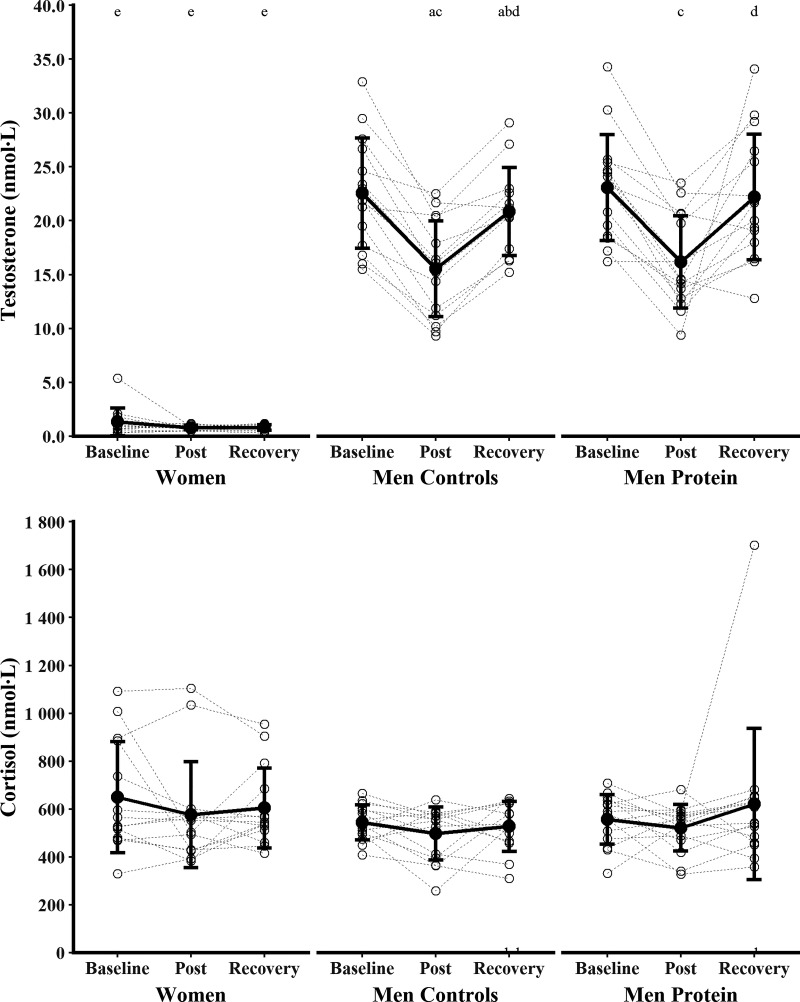
Testosterone and cortisol before (baseline), 24 h after (post), and 96 h after (recovery) the field exercise. Women (*n* = 14) and men supplemented with protein (Men Protein, *n* = 15) were independently compared with nonsupplemented men (Men Controls, *n* = 15) to examine the effect of sex and protein supplementation. Data were analyzed with linear mixed-effects models. ^a^*P* < 0.05 vs. baseline (within group); ^b^*P* < 0.05 vs. post (within group); ^c^*P* < 0.05 vs. baseline (main effects, Men Controls and Men Protein pooled); ^d^*P* < 0.05 vs. post (main effects, Men Controls and Men Protein pooled); ^e^*P* < 0.05 vs. Men Controls (main effect of sex).

Biochemical markers of bone metabolism and calcium metabolism are presented in Figs. 3, 4, and 5 with mean absolute differences presented in Table 3. βCTX, total 1,25(OH)_2_D, and cortisol were not different between time points (main effects of time, *P* ≥ 0.094, η_p_^2^ ≤ 0.09) or Women and Men Controls (main effect of sex, *P* ≥ 0.069, η_p_^2^ ≤ 0.12; sex × time interaction, *P* ≥ 0.245, η_p_^2^ ≤ 0.05). There were main effects of time for PINP, PTH, albumin-adjusted calcium, phosphate, total 25(OH)D, and total 24,25(OH)_2_D (*P* < 0.005, η_p_^2^ ≥ 0.18), but no difference between Women and Men Controls (main effects of sex, *P* ≥ 0.122, η_p_^2^ ≤ 0.09; sex × time interactions, *P* ≥ 0.125, η_p_^2^ ≤ 0.08). PINP decreased from baseline to postexercise (*P* < 0.001, *g* = 1.52) and recovery (*P* < 0.001, *g* = 0.68), with postexercise lower than recovery (*P* = 0.010, *g* = 0.52). PTH increased from baseline to postexercise (*P* = 0.006, *g* = 0.63) and decreased from postexercise to recovery (*P* = 0.047, *g* = 0.44), with no difference between baseline and recovery (*P* = 1.000, *g* = 0.12). Albumin-adjusted calcium increased from baseline to recovery (*P* = 0.006, *g* = 0.54) and from postexercise to recovery (*P* < 0.001, *g* = 0.98), but baseline and postexercise were not different (*P* = 0.434, *g* = 0.27). Phosphate increased from postexercise to recovery (*P* = 0.001, *g* = 0.67), but baseline and postexercise (*P* = 0.082, *g* = 0.46) and baseline and recovery were not different (*P* = 0.369, *g* = 0.27). Total 25(OH)D increased from baseline to postexercise (*P* = 0.038, *g* = 0.45) and recovery (*P* < 0.001, *g* = 0.96), and from postexercise to recovery (*P* = 0.016, *g* = 0.59). Total 24,25(OH)_2_D decreased from baseline to recovery (*P* = 0.011 *g* = 0.51), but baseline and postexercise (*P* = 0.100, *g* = 0.43) and postexercise and recovery (*P* = 1.000, *g* = 0.16) were not different. There was a sex × group interaction for testosterone (*P* < 0.001, η_p_^2^ = 0.52). Testosterone decreased from baseline to postexercise (*P* < 0.001, *g* = 1.97) and recovery (*P* = 0.007, *g* = 0.50), and increased from postexercise to recovery (*P* < 0.001, *g* = 2.05), in Men Controls. Testosterone did not change for Women at any time point (all *P* = 1.000, *g* ≤ 0.41). Testosterone was lower in Women than in Men Controls at all time points (all *P* < 0.001, *g* ≥ 4.48).

**Table 3.** Mean absolute changes [95% confidence intervals] of biochemical markers of bone formation, bone resorption, and calcium metabolism

	Baseline vs. Post Exercise	Baseline vs. Recovery	Post Exercise vs. Recovery
Women			
βCTX, μg·L^−1^	0.05 [−0.02, 0.13]	0.07 [−0.01, 0.16]	0.02 [−0.08, 0.12]
PINP, μg·L^−1^	−11.0 [−15.9, −6.1]	−4.9 [−11.5, 1.6]	6.1 [−1.4, 13.6]
PTH, pmol·L^−1^	0.6 [0.0, 1.3]	0.2 [−0.5, 0.8]	−0.5 [−1.1, 0.2]
Adjusted calcium, mmol·L^−1^	−0.01 [−0.04, 0.02]	0.05 [0.01, 0.09]	0.06 [0.03, 0.09]
Phosphate, mmol·L^−1^	−0.11 [−0.21, 0.00]	0.05 [−0.07, 0.18]	0.16 [0.04, 0.28]
Total 25(OH)D, nmol·L^−1^	3.2 [−1.0, 7.4]	11.7 [7.3, 16.1]	8.5 [4.9, 12.1]
Total 1,25(OH)_2_D, nmol·L^−1^	0.7 [−16.2, 17.6]	−9.3 [−25.0, 6.4]	−10.0 [−24.5, 4.4]
Total 24,25(OH)_2_D, nmol·L^−1^	−1.2 [−1.7, −0.7]	−0.8 [−1.9, 0.2]	0.4 [−0.7, 1.5]
Testosterone, nmol·L^−1^	−0.5 [−1.2, 0.2]	−0.5 [−1.2, 0.2]	0.0 [−0.1, 0.1]
Cortisol, nmol·L^−1^	−73 [−182, 35]	−46 [−144, 52]	28 [−56, 111]
Men Controls			
βCTX, μg·L^−1^	0.00 [−0.07, 0.07]	0.04 [−0.02, 0.09]	0.03 [−0.06, 0.13]
PINP, μg·L^−1^	−15.9 [−20.6, −11.2]	−10.4 [−16.3, 4.6]	5.6 [−1.4, 13.6]
PTH, pmol·L^−1^	0.7 [0.2, 1.3]	0.1 [−0.6, 0.8]	−0.6 [−1.4, 0.2]
Adjusted calcium, mmol·L^−1^	−0.02 [−0.06, 0.01]	0.02 [−0.02, 0.05]	0.04 [0.01, 0.07]
Phosphate, mmol·L^−1^	−0.04 [−0.10, 0.02]	0.05 [−0.05, 0.15]	0.09 [0.00, 0.18]
Total 25(OH)D, nmol·L^−1^	5.7 [−0.8, 12.4]	7.2 [0.4, 14.0]	2.2 [−4.1, 8.6]
Total 1,25(OH)_2_D, nmol·L^−1^	−5.5 [−17.2, 6.2]	−0.3 [−12.1, 11.4]	5.0 [−7.0, 17.0]
Total 24,25(OH)_2_D, nmol·L^−1^	−0.3 [−1.5, 0.9]	−1.4 [−2.9, 0.1]	−1.1 [−2.1, 0.1]
Testosterone, nmol·L^−1^	−7.0 [−8.9, −5.2]	−2.2 [−4.7, 0.3]	4.9 [3.5, 6.2]
Cortisol, nmol·L^−1^	−46 [−106, 13]	−14 [−81, 53]	34 [−47, 115]
Men Protein			
βCTX, μg·L^−1^	−0.09 [−0.15, −0.03]	0.01 [−0.05, 0.06]	0.10 [0.05, 0.14]
PINP, μg·L^−1^	−13.1 [−17.4, −8.8]	−9.1 [−12.7, 5.5]	4.1 [0.5, 7.6]
PTH, pmol·L^−1^	0.3 [−0.3, 0.8]	−0.3 [−0.9, 0.2]	−0.6 [−1.2, 0.0]
Adjusted calcium, mmol·L^−1^	0.02 [−0.01, 0.05]	0.06 [0.02, 0.10]	0.04 [0.01, 0.08]
Phosphate, mmol·L^−1^	0.03 [−0.05, 0.12]	0.01 [−0.08, 0.11]	−0.02 [−0.10, 0.06]
Total 25(OH)D, nmol·L^−1^	1.0 [−4.7, 6.7]	4.9 [−1.7, 11.5]	3.9 [−1.4, 9.2]
Total 1,25(OH)_2_D, nmol·L^−1^	−9.8 [−25.4, 5.8]	7.1 [−4.1, 18.3]	16.9 [5.3, 28.5]
Total 24,25(OH)_2_D, nmol·L^−1^	−1.0 [−2.1, 0.0]	−1.0 [−2.3, 0.3]	0.0 [−1.3, 1.3]
Testosterone, nmol·L^−1^	−6.9 [−8.9, −4.9]	−0.9 [−4.8, 3.0]	6.0 [2.4, 9.6]
Cortisol, nmol·L^−1^	−36 [−105, 34]	64 [−122, 249]	99 [−64, 263]

βCTX, beta C-telopeptide cross-links of type 1 collagen; PINP, procollagen I N-terminal propeptide; PTH, parathyroid hormone; total 25(OH)D, total 25-hydroxyvitamin D; total 1,25(OH)_2_D, total 1,25-dihydroxyvitamin D; total 24,25(OH)_2_D, total 24,25-dihydroxyvitamin D.

### The Effect of Protein Supplementation on Biochemical Markers of Bone Resorption, Bone Formation, and Calcium Metabolism

Biochemical markers of bone metabolism and calcium metabolism are presented in Figs. 3, 4, and 5 with mean absolute differences presented in Table 3. There were main effects of time for βCTX, PINP, PTH, albumin-adjusted calcium, total 25(OH)D, total 1,25(OH)_2_D, total 24,25(OH)_2_D, and testosterone (*P* ≤ 0.023, η_p_^2^ ≥ 0.13), but no effect of protein supplementation (main effects of group, *P* ≥ 0.111, η_p_^2^ ≤ 0.09; group × time interactions, *P* ≥ 0.084, η_p_^2^ ≤ 0.09). βCTX did not change from baseline to postexercise (*P* = 0.089, *g* = 0.36) or recovery (*P* = 0.899, *g* = 0.20), but increased between postexercise and recovery (*P* = 0.007, *g* = 0.55). PINP decreased from baseline to postexercise (*P* < 0.001, *g* = 1.75) and recovery (*P* < 0.001, *g* = 1.18) and increased between postexercise and recovery (*P* = 0.006, *g* = 0.62). PTH increased from baseline to postexercise (*P* = 0.048, *g* = 0.47) and decreased from postexercise to recovery (*P* = 0.015, *g* = 0.50), with baseline and recovery not different (*P* = 1.000, *g* = 0.12). Albumin-adjusted calcium increased from baseline to recovery (*P* = 0.002, *g* = 0.57) and from postexercise to recovery (*P* = 0.001, *g* = 0.71), but baseline and postexercise were not different (*P* = 1.000, *g* = 0.03). Total 25(OH)D increased from baseline to recovery (*P* = 0.010, *g* = 0.51), but baseline and postexercise (*P* = 0.289, *g* = 0.29) and postexercise and recovery (*P* = 0.469, *g* = 0.31) were not different. Total 1,25(OH)_2_D increased from postexercise to recovery (*P* = 0.024, *g* = 0.53), but baseline and postexercise (*P* = 0.181, *g* = 0.30) and baseline and recovery (*P* = 1.000, *g* = 0.18) were not different. Total 24,25(OH)_2_D decreased from baseline to recovery (*P* = 0.018, *g* = 0.49), but baseline and postexercise (*P* = 0.301, *g* = 0.32) and postexercise and recovery (*P* = 0.666, *g* = 0.23) were not different. Testosterone decreased from baseline to postexercise (*P* < 0.001, *g* = 1.96) and increased from postexercise to recovery (*P* < 0.001, *g* = 1.08), but baseline and recovery were not different (*P* = 0.351, *g* = 0.25). Phosphate and cortisol were not different between time points (main effects of time, *P* ≥ 0.244, η_p_^2^ ≤ 0.05) or groups (main effect of group, *P* ≥ 0.259, η_p_^2^ ≤ 0.04; group × time interaction, *P* ≥ 0.144, η_p_^2^ ≤ 0.07).

## DISCUSSION

A 36-h field exercise involving ∼70 km of load carriage carrying 25 kg, ≤4 h of total sleep, and a severe energy deficit (∼2,000–3,000 kcal·day^−1^) decreased PINP and increased PTH in women and men, decreased testosterone in men, and had no effect on βCTX. Men supplemented with protein consumed ∼50 g·day^−1^ more protein and ∼900 kcal·day^−1^ more energy than men consuming the habitual diet, but protein supplementation had no effect on any metabolic marker. Although there are data examining the bone metabolic response to several months of basic military training in female and male recruits (24–30) and 8-wk specialist combat training courses in trained male soldiers (8, 10), there are no data examining acute responses to short periods of military operational stress. Women have recently been allowed to enter UK Armed Forces combat roles alongside men, but there is a lack of data on women in response to the physiological stressors associated with combat—high levels of physical of activity, energy deficiency, and sleep deprivation (1, 15). The data in this study provide new insight into the suppression of a metabolic marker of bone formation in both men and women in response to an acute period of extreme exercise and nutritional stress.

### Biochemical Markers of Bone Resorption and Bone Formation

We observed no change in βCTX—a measure of type I collagen degradation—in the comparison of women and men. There was an increase in βCTX between postexercise and recovery in men (pooled analysis of Men Controls and Men Protein). It is not clear if this increased βCTX between postexercise and recovery is because of suppressed βCTX immediately after the field exercise or increased βCTX following recovery. Prolonged moderate-intensity running has been shown to decrease βCTX (31) and could explain suppressed βCTX immediately after the field exercise, but high-intensity or exhaustive running had no effect (31) or increased βCTX (32, 33). Exercise mode appears to influence the βCTX response, with low-impact prolonged aerobic activities generally causing the biggest increases (17). Short periods of low energy availability (5 days) increased βCTX in women (11, 12). A 61-day Antarctic traverse with severe energy deficit (∼13% body mass loss) had no effect on βCTX in Servicewomen; however, the sample size was small, measures were taken after 4 days of recovery feeding, and there were large effect sizes for increased βCTX (34). Our sample size was determined to detect an effect size (sex × interaction) of η_p_^2^ = 0.04 (small effect). Sensitivity power analysis revealed that our study was actually able to detect any effect size (sex × interaction) of η_p_^2^ ≥ 0.05 with 80% power, but our observed effect size for βCTX was η_p_^2^ = 0.02. Our βCTX findings could therefore be type II error; however, any effect is likely to be small. Our data do not provide sufficient evidence for increased bone resorption in response to a short military field exercise in energy deficit, or a difference between women and men. The duration of the field exercise was short, and 24 h of energy deficit did not have any effect on βCTX in men or women in a laboratory trial (35). The βCTX response to longer periods of military training is complex with decreased (8, 29, 36), increased (25, 26, 28), and unchanged (10, 30) βCTX reported in military training studies of 8–16 wk in men and/or women. Some of these studies also report adaptive bone formation at the tibia, demonstrating a complex relationship between βCTX and skeletal adaptation (27–30, 36). One study reported similar increases in βCTX between sexes during 16 wk of basic military training (25), and another study reported no effect of protein supplementation on βCTX during 9 wk basic military training (21), supporting our findings that the bone resorption response to military activities does not differ between women and men and is not influenced by an additional protein intake of ∼50 g·day^−1^. The lack of effect of protein supplementation must be interpreted with caution as the control group still consumed a high amount of protein (122 ± 35 g·day^−1^ or 1.6 ± 0.5 g·kg·day^−1^).

Procollagen type I N-terminal propeptide—a measure of type I collagen synthesis—decreased from baseline to postexercise and recovery. The PINP data suggest that a short period of military field exercise suppressed bone formation, which remained lower than baseline following 96 h of ad libitum food intake and recovery. The PINP response was not different between men and women and was not protected by an additional intake of ∼50 g·day^−1^ protein supplementation for men. The observed sex × interaction effect size for PINP was small (η_p_^2^ = 0.05) and any effect was not detectable with our statistical power. Laboratory studies show that 5-day low energy availability decreased PINP production in women and men, with no difference between sexes (12), but ≥ 60 min treadmill running had no effect on (32) or increased (31, 33) PINP production. Acute exercise typically increases markers of bone formation (17), and therefore the decrease in PINP production was likely due to energy deficiency, although 24 h of energy restriction had no effect on PINP in men or women in another laboratory trial (35), and acute periods of sleep deprivation can also decrease bone formation (16). Women were in a smaller absolute energy deficit compared with men (∼2,000 kcal vs. 2,900 kcal·day^−1^), and so women may experience disturbances in bone formation at lower severities of energy deficits. The PINP response to military training in energy deficit is inconsistent; PINP was unchanged in women following severe energy deficit during a 61-day Antarctic crossing (34) and increased in men during 8-wk combat training in moderate energy deficit (∼500 kcal·day^−1^) (8). Other studies have reported decreased bone-specific alkaline phosphatase (bone ALP) following 8-wk military combat courses in energy deficits (∼500–1,000 kcal·day^−1^) (8, 10), but PINP and bone ALP represent different bone formation processes with different responses to training and nutrition (8) and so comparisons between markers should be made with caution. Basic military training studies report increased (25, 26) or unchanged (27–30, 36) PINP production in men and women over 8–16 wk, alongside adaptive bone formation at the tibia (27–30, 36). The increase in PINP during 16 wk of basic military training was similar between men and women (25), and protein supplementation had no effect on PINP during 9 wk of basic military training (21). We similarly observed no evidence of a sex difference when PINP production was decreased by a military field exercise and no protective effect of protein supplementation. The implications for acute decreases in type I collagen formation for stress fracture risk and adaptive bone formation are unclear, but a high incidence of stress fractures (1.9% for men, 11.4% for women) has been reported during this training course (37).

### Biochemical Markers of Calcium Metabolism

Parathyroid hormone increased 24 h after the field exercise compared with baseline and decreased between postexercise and recovery. The observed sex × interaction effect size for PTH was very small (η_p_^2^ < 0.01). There was no sex difference and no effect of protein supplementation on the PTH response. Increases in PTH have previously been reported after several months of basic military training (26, 27), although decreased (25) and unchanged (10, 29, 36) PTH have also been shown in men and women. Parathyroid hormone secretion is regulated by serum ionized calcium (38) and phosphate (39), and PTH mobilizes skeletal calcium by stimulating bone resorption (38). The increase in PTH was not accompanied by an increase in βCTX, but the anabolic and catabolic actions of PTH are complex (38). Our study design makes it challenging to identify the mechanisms for increases in PTH as PTH increases within minutes following a decrease in serum-ionized calcium and changes in serum-ionized calcium and phosphate are both causes and consequences of changes in PTH. Albumin-adjusted calcium—an estimate of ionized calcium—and phosphate were not different from baseline after the field exercise and so the direct mechanism for the increase in PTH is unclear. Exercise acutely decreases ionized calcium and increases phosphate resulting in increased PTH production (40, 41), although an increase in PTH only occurs when the exercise intensity is high (31) or the exercise is prolonged (38). The demands of British Army military training are typically higher for women than for men (42), which might explain our previous finding that PTH increased in women but not in men (24). The field exercise in this study was high-intensity and prolonged for both men and women as evidenced by the high total energy expenditures, which may have masked any sex differences in the PTH response. Parathyroid hormone secretion follows a circadian rhythm, which is also disturbed by sleep disturbances and fasting (43), and so our PTH changes may represent a shift in this circadian rhythm. The implications of an increase in PTH for stress fracture risk and adaptive bone formation are not clear; intermittent increases in PTH are osteogenic (38) yet higher PTH has been associated with increased stress fracture risk (44). Previous studies showed a higher protein diet (2.1 g·day^−1^ vs. 1.0 g·day^−1^) increased intestinal calcium absorption (45) and a lower protein diet (0.7 g·day^−1^ vs. 1.0 g·day^−1^) decreased PTH (46), although increasing dietary protein intake during energy deficit (from 0.8 g·day^−1^ to 1.6 g·day^−1^ or 2.4 g·day^−1^) had no effect on calcium absorption or PTH (47). The protein supplement in our study did not influence markers of calcium metabolism likely because of the greater contribution of high-intensity and prolonged exercise on disruptions to PTH, but also potentially because of the high volume of protein consumed in the control group.

Total 25(OH)D increased from baseline to postexercise and from postexercise to recovery, with no difference between women and men, and no effect of protein supplementation. The increase in total 25(OH)D was high (5–12 nmol·L^−1^ depending on group) in the short time frame in this study. The mechanism is likely an increase in fat oxidation with prolonged exercise and energy deficit (48). An increase in total 25(OH)D could have contributed to the decreased PTH from postexercise to recovery and increased calcium and phosphate in recovery. The active 25(OH)D metabolite 1,25(OH)_2_D contributes to calcium and phosphate homeostasis by providing negative feedback of PTH secretion (38) and increasing calcium and phosphate absorption from the gastrointestinal tract (39). Total 1,25(OH)_2_D was unchanged, which is unsurprising considering the tight regulation of 1,25(OH)_2_D independently of total 25(OH)D concentrations (49). An increase in total 25(OH)D coincided with a decrease in total 24,25(OH)_2_D from baseline to recovery, which is in contrast to the positive linear relationship between 25(OH)D and 24,25(OH)_2_D and could be due to disturbances to the hydroxylase enzymes (49). Unchanged total 1,25(OH)_2_D and decreased total 24,25(OH)_2_D increase the ratio between these two metabolites (vitamin D metabolite ratio) (49). The implications of changes in 24,25(OH)_2_D are not clear, but higher vitamin D metabolite ratios are associated with poorer physical performance (50) and higher PTH (49). These data present a novel analysis of changes in vitamin D metabolites following acute physiological stress.

### Reproductive and Adrenal Hormones

Testosterone decreased from baseline to postexercise and recovery and increased from postexercise to recovery in men. Military training in energy deficit has consistently been shown to decrease testosterone in men over training courses ranging from several days to 8 wk (2–6, 9, 51), but to our knowledge, this study provides the first evidence that a military field exercise as short as 36 h can decrease testosterone. The sex steroids testosterone and estradiol are important regulators of bone metabolism (52). Testosterone can have a direct effect on bone through the androgen receptor, but estradiol is the main regulator of bone metabolism in men through peripheral aromatization of testosterone (52). Estradiol suppresses osteoclast activity (53) and low concentrations of estradiol with energy deficiency increase bone resorption in physically active women (11). The effect of energy restriction on sex steroid concentrations and bone in men is less well understood, but here we observed low testosterone and decreased PINP in men. We observed no change in bone resorption despite decreased testosterone, although we did not measure free testosterone or estradiol and increases in sex hormone binding globulin are observed after arduous military training courses in energy deficits decreasing free testosterone and estradiol (3, 6, 8). The decrease in bone formation may also be due to a decrease in IGF-I and/or other alterations to the IGF axis caused by energy deficiency (8). We did not measure IGF-I or the IGF-binding proteins in this study, but IGF-I is an important regulator of bone formation (54), and military training has consistently shown to decrease IGF-I and alter concentrations of the binding proteins, even after just several days (3–8). Cortisol was not different across time points in either men or women (sex × interaction, η_p_^2^ < 0.01) and so was unlikely to contribute to decreased bone formation.

The few military training studies that have provided supplementary energy found no protective effect on sex steroid concentrations (2, 3, 8, 51), consistent with our data. Increasing protein intake to 2 g·kg^−1^·day^−1^ during a 10-day military field exercise in energy deficit did not protect the disturbances to testosterone, thyroid hormones, or IGF-I compared with the habitual ration packs (1 g·kg^−1^·day^−1^) (19). Although 0.9 g·kg^−1^·day^−1^ of protein intake attenuated a decrease in IGF-I compared with 0.5 g·kg^−1^·day^−1^ of protein intake, there were no effects of increased protein intake on other parts of the IGF-I axis or testosterone (20). A randomized controlled trial showed that increasing protein intake to two or three times the recommended daily allowance during a 40% energy deficit had no effect on endocrine markers, calcium absorption or metabolism, or bone metabolism (20, 47). Supplementary protein had no protective effect on testosterone in our study, and these previous studies, likely because the additional energy was insufficient to eliminate the energy deficit, or mechanisms other than energy deficiency such as sleep restriction or high levels of physical activity, were responsible for the reduction in testosterone.

### Limitations

The findings in this study are limited by the small sample size, the limited number of time points captured, and the short study duration, which likely meant some of our outcomes were underpowered or some effects were undetectable with our study design. Sensitivity power analysis revealed that our study was able to detect any sex × interaction effect size of η_p_^2^ ≥ 0.05 (small effects) with 80% power, and so our study would have only been underpowered for detecting small effects and the impact of any type II error on our conclusions would have been minimal. Our postexercise measures were taken 24 h after the field exercise and so acute changes in our markers may have been missed. The low numbers of women going through British Army Officer training meant we were unable to include a group of women supplemented with protein. We were also unable to blind the control group, but do not believe the unblinded nature of the trial impacted the results. We did not measure estradiol, sex hormone binding globulin, or IGF-I, which may have helped in the interpretation of the bone metabolism data. However, the measurement and interpretation of estradiol over the time frame in this study were unfeasible and lacked external validity as some of the women took a range of hormonal contraceptives and others were at different stages of the menstrual cycle. We did not adjust our circulating measures of bone metabolism for potential changes in plasma volume. Finally, we did not have a measure of calcium or phosphate intake; calcium may interact with protein to increase calcium intestinal absorption, and phosphate intake is important in the circadian rhythm of PTH.

### Conclusions

A 36-h field exercise suppressed a marker of bone formation for 4 days in men and women, with no difference between sexes. Protein supplementation had no protective effect on the decrease in bone formation or testosterone. The mechanism for this decrease in bone formation is unclear but could be due to the acute effects of low energy availability on metabolic regulators of bone metabolism. The implications of acute decreased bone formation for skeletal adaptations and stress fracture risk warrant further investigation.

## DATA AVAILABILITY

Data will be made available upon reasonable request.

## GRANTS

This study was funded by the UK Ministry of Defense (Army).

## DISCLOSURES

No conflicts of interest, financial or otherwise, are declared by the authors.

## AUTHOR CONTRIBUTIONS

T.J.O., V.C.E., S.L.W., and J.P.G. conceived and designed research; T.J.O., C.V.C., V.C.E., R.L.K., F.N.K., J.C.Y.T., W.D.F., and S.L.W. performed experiments; T.J.O. analyzed data; T.J.O., C.V.C., S.L.W., and J.P.G. interpreted results of experiments; T.J.O. prepared figures; T.J.O. drafted manuscript; T.J.O., C.V.C., V.C.E., S.D.B., R.L.K., J.C.Y.T., W.D.F., S.L.W., and J.P.G. edited and revised manuscript; T.J.O., C.V.C., V.C.E., S.D.B., R.L.K., F.N.K., J.C.Y.T., W.D.F., S.L.W., and J.P.G. approved final version of manuscript.
